# Supplementary material for: Dynalign II: common secondary structure prediction for RNA homologs with domain insertions
Source: Nucleic Acids Res. 2014 Nov 21;42(22):13939–48. doi: 10.1093/nar/gku1172 (PMC4267632; doi:10.1093/nar/gku1172)
Supplement: SUPPLEMENTARY DATA [file supp_gku1172_nar-02021-z-2014-File012.zip › manual/javadoc/RNAstructure_java_utilities/ImageGrabber.html]

ImageGrabber


JavaScript is disabled on your browser.


- Overview
- Package
- Class
- Tree
- Index
- Help

- Prev Class
- Next Class

- Frames
- No Frames

- All Classes

- Summary:
- Nested |
- Field |
- Constr |
- Method

- Detail:
- Field |
- Constr |
- Method


RNAstructure\_java\_utilities

## Class ImageGrabber

- java.lang.Object
- - RNAstructure\_java\_utilities.ImageGrabber

- All Implemented Interfaces:
  :   Serializable

  ---

    

  ```
  public class ImageGrabber
  extends Object
  implements Serializable
  ```

  A class that creates images in various contexts for the RNAstructure GUI.

  See Also:
  :   Serialized Form

- - ### Field Summary

    Fields

    | Modifier and Type | Field and Description |
    | --- | --- |
    | `private static long` | `serialVersionUID` |
  - ### Constructor Summary

    Constructors

    Constructor and Description

    `ImageGrabber()`
  - ### Method Summary

    Methods

    | Modifier and Type | Method and Description |
    | --- | --- |
    | `static BufferedImage` | `getImage(String path)` Create an image. |
    | `static ImageIcon` | `getImageIcon(String path)` Create an image icon. |
    | `static JLabel` | `getImageLabel(String path)` Create an image label. |

    - ### Methods inherited from class java.lang.Object

      `clone, equals, finalize, getClass, hashCode, notify, notifyAll, toString, wait, wait, wait`

- - ### Field Detail


    - #### serialVersionUID

      ```
      private static final long serialVersionUID
      ```

      See Also:
      :   Constant Field Values
  - ### Constructor Detail


    - #### ImageGrabber

      ```
      public ImageGrabber()
      ```
  - ### Method Detail


    - #### getImage

      ```
      public static BufferedImage getImage(String path)
                                    throws IOException
      ```

      Create an image.

      Parameters:
      :   `path` - The path to the image.

      Returns:
      :   The image.

      Throws:
      :   `IOException` - If an image cannot be read correctly. Since all
          image paths are hard coded, this should never
          be thrown.


    - #### getImageIcon

      ```
      public static ImageIcon getImageIcon(String path)
                                    throws IOException
      ```

      Create an image icon.

      Parameters:
      :   `path` - The path to the image.

      Returns:
      :   The image icon.

      Throws:
      :   `IOException` - If an image cannot be read correctly. Since all
          image paths are hard coded, this should never
          be thrown.


    - #### getImageLabel

      ```
      public static JLabel getImageLabel(String path)
                                  throws IOException
      ```

      Create an image label.

      Parameters:
      :   `path` - The path to the image.

      Returns:
      :   The image label.

      Throws:
      :   `IOException` - If an image cannot be read correctly. Since all
          image paths are hard coded, this should never
          be thrown.


- Overview
- Package
- Class
- Tree
- Index
- Help

- Prev Class
- Next Class

- Frames
- No Frames

- All Classes

- Summary:
- Nested |
- Field |
- Constr |
- Method

- Detail:
- Field |
- Constr |
- Method
